# Human Locomotion in Hypogravity: From Basic Research to Clinical Applications

**DOI:** 10.3389/fphys.2017.00893

**Published:** 2017-11-07

**Authors:** Francesco Lacquaniti, Yury P. Ivanenko, Francesca Sylos-Labini, Valentina La Scaleia, Barbara La Scaleia, Patrick A. Willems, Myrka Zago

**Affiliations:** ^1^Department of Systems Medicine, University of Rome Tor Vergata, Rome, Italy; ^2^Center of Space BioMedicine, University of Rome Tor Vergata, Rome, Italy; ^3^Laboratory of Neuromotor Physiology, IRCCS Santa Lucia Foundation, Rome, Italy; ^4^Laboratory of Biomechanics and Physiology of Locomotion, Institute of NeuroScience, Université catholique de Louvain, Louvain-la-Neuve, Belgium

**Keywords:** human locomotion, body weight support, hypogravity simulators, moon walk, parabolic flight, locomotion rehabilitation, robotic gravity-assist

## Abstract

We have considerable knowledge about the mechanisms underlying compensation of Earth gravity during locomotion, a knowledge obtained from physiological, biomechanical, modeling, developmental, comparative, and paleoanthropological studies. By contrast, we know much less about locomotion and movement in general under sustained hypogravity. This lack of information poses a serious problem for human space exploration. In a near future humans will walk again on the Moon and for the first time on Mars. It would be important to predict how they will move around, since we know that locomotion and mobility in general may be jeopardized in hypogravity, especially when landing after a prolonged weightlessness of the space flight. The combination of muscle weakness, of wearing a cumbersome spacesuit, and of maladaptive patterns of locomotion in hypogravity significantly increase the risk of falls and injuries. Much of what we currently know about locomotion in hypogravity derives from the video archives of the Apollo missions on the Moon, the experiments performed with parabolic flight or with body weight support on Earth, and the theoretical models. These are the topics of our review, along with the issue of the application of simulated hypogravity in rehabilitation to help patients with deambulation problems. We consider several issues that are common to the field of space science and clinical rehabilitation: the general principles governing locomotion in hypogravity, the methods used to reduce gravity effects on locomotion, the extent to which the resulting behavior is comparable across different methods, the important non-linearities of several locomotor parameters as a function of the gravity reduction, the need to use multiple methods to obtain reliable results, and the need to tailor the methods individually based on the physiology and medical history of each person.

## Introduction

Human missions are considered vital for harvesting the maximum benefits from space exploration (White and Averner, 2001). However, space travelers are exposed to several challenges and risks, ranging from radiation to isolation and altered gravity effects. Here we are concerned specifically with the effects of reduced gravity (hypogravity, 0 < g < 1) on locomotion, such as it would be experienced on the Moon or Mars. In this regards, a recent survey (White et al., 2016) has remarked that, while the effects of sustained weightlessness (0 g) on sensory and motor functions have been rather extensively investigated, much less is known about the effects of sustained hypogravity on these functions. White et al. (2016) further remarked that the transient and adaptive effects on sensory-motor functions have been well-studied following transitions from 0 to 1 g (and, to a lesser extent, from 1 to 0 g), whereas little is known about the transitions from 1 g to hypo-g and vice versa.

Examination of the records from previous space flights, especially at low-Earth orbits, revealed that astronauts often suffer from several problems related to the locomotor system, from osteoporosis to muscle atrophy, changes of tendons elasticity and altered neural control of posture and movement (Bloomberg et al., 2016; White et al., 2016; Lang et al., 2017). These problems are exacerbated after long-duration missions. Moreover, landing on the Moon or Mars will further challenge the motor control system because of the sudden transition from the weightlessness of the space travel to the restored albeit reduced gravity of the Moon (0.16 g) or Mars (0.38 g). In such circumstances, motor problems may lead to disastrous outcomes in an environment with limited or no medical assistance. A simple fall due to muscle weakness or impaired control of posture and movement can lead to muscle strains, ligament sprains, bone fractures, head traumas, or other more or less severe injuries that would be very difficult to treat in an alien environment. Damages to the spacesuit or the portable life support system resulting from a fall may also be life-threatening. These and related issues have been brought to the attention of the space biomedical community by NASA (see NASA Roadmap for Human Health Risks), ESA, and EC (see THESEUS roadmap). It has been remarked that no existing countermeasure is deemed sufficient to reduce some of these risks to an acceptable level.

The main current countermeasure for preventing disorders of the locomotor apparatus in space is represented by daily, intensive exercises, especially walking and running on a treadmill. Unfortunately, these exercises prevent the motor problems only to a limited extent. Thus, upon return to Earth, most astronauts exhibit significant loss of bone and muscle tissue, as well as a significant reduction of mobility (Mulavara et al., 2010). We believe that the limited success of current countermeasures is due to the lack of sufficient knowledge of the impact of reduced gravity on the motor system, and therefore to the inadequate design of countermeasures including exercise devices and protocols for the astronauts.

A different but related topic is represented by the application of space research to medicine on Earth. There are two aspects to be considered. First, understanding how astronauts adapt to and recover from micro- or hypo-gravity could also benefit patients on Earth who exhibit a decline in motor performance due to a disabling disease or prolonged bed-rest that limited the effects of gravity on their movements. Second, a simulation of reduced gravity—such as with body weight unloading—is used more and more in rehabilitation to help patients with motor disorders. Therefore, a better understanding of the physiological adaptation of locomotor control to hypogravity may help designing more effective simulators for rehabilitation.

Here we first review the basic principles underlying locomotion under gravity, including some developmental and evolutionary issues, then we describe the locomotor behavior that has been monitored in actual hypogravity (Moon, parabolic flight) and simulated hypogravity (Earth laboratory). Finally, we examine current assessments of the therapeutical efficacy of body weight unloading in several pathologies. Specific consideration will be given to the methodologies for simulating hypogravity in the laboratory for research and in clinical environments for rehabilitation.

The present article aims at providing an update relative to the previous reviews on similar topics (e.g., Davis and Cavanagh, 1993; Lackner and DiZio, 1996, 2000; Reschke et al., 1998; Newman, 2000; Bloomberg and Mulavara, 2003; Clément et al., 2005; Clément and Reschke, 2008; Sylos-Labini et al., 2014b; White et al., 2016). Because of space limitations, we consider a number of hypogravity conditions relevant to locomotion but we exclude water immersion, which allows only limited locomotion in humans (e.g., Duddy, 1969; Watenpaugh, 2016). Moreover, we focus on the motion of the center of body mass (COM) and limbs, while for gaze and postural control in hypogravity the reader may want to consult Bloomberg et al. (1997), Lackner and DiZio (2000), Mulavara and Bloomberg (2002), Clément and Reschke (2008), Mulavara et al. (2012).

## Gravity effects on locomotion

**Figure 1:**
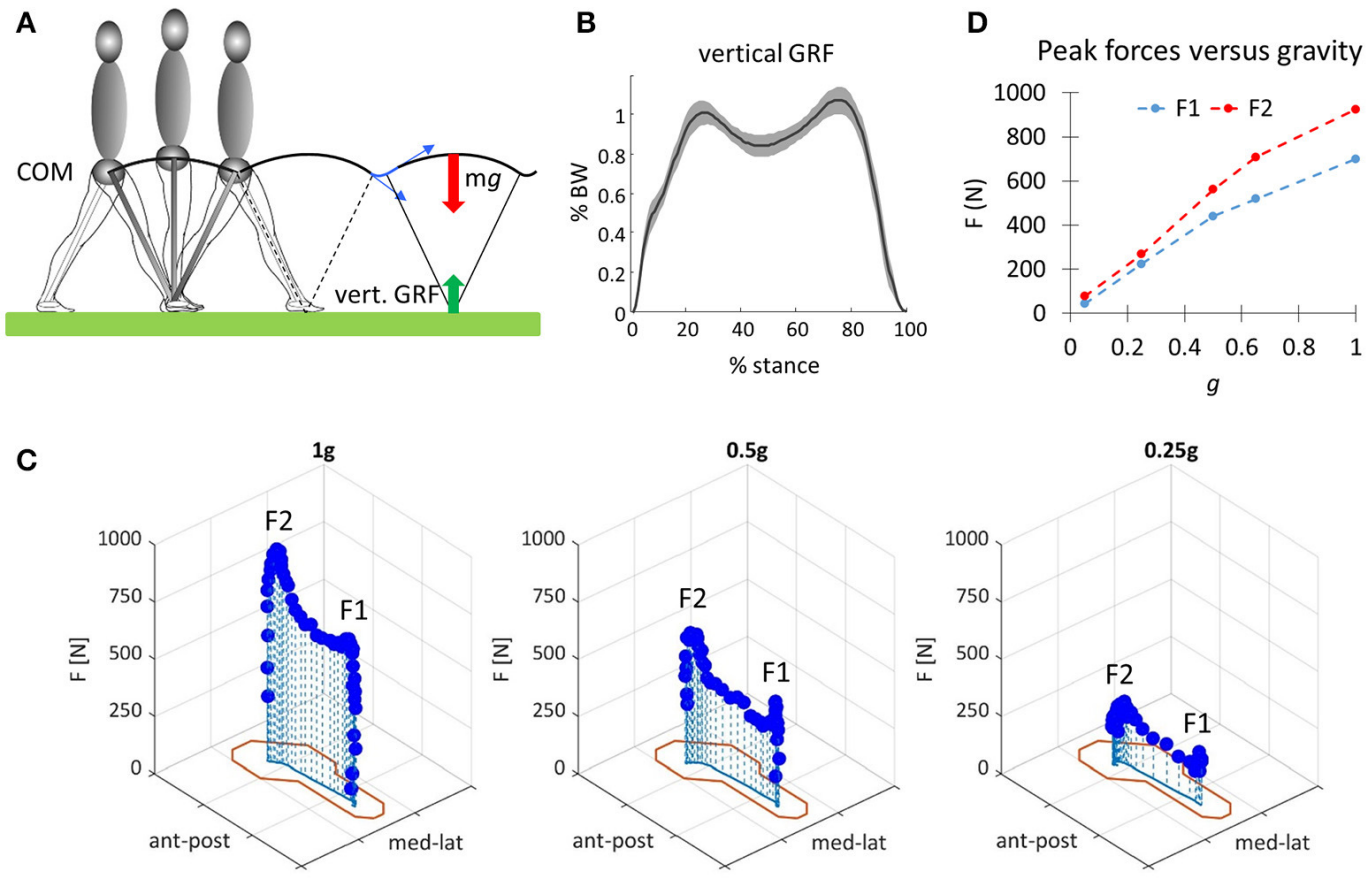
**(A)** Schematic illustration of the pendulum-like behavior of the center-of-body mass (COM) during walking. Red and green arrows denote body weight and vertical ground reaction force (GRF), respectively. Blue arrows denote COM velocity before and after heel contact (step-to-step transitions). **(B)** Ensemble average (±*SD*, 20 subjects, normalized to individual body weight BW) of the time profile of the vertical ground reaction force (GRF, recorded with a force plate) plotted vs. normalized stance duration. Data replotted from Martino et al. (2014). **(C)** Changes of the vertical component of in-shoe reaction forces plotted as a function of the spatial coordinates of the foot (outline in red corresponding to the outer elements of the pressure-sensitive matrix interposed between foot and shoe) at three different levels of gravity obtained in the laboratory with a vertical body-weight supporting (BWS) system: 1 g (0 BWS), 0.5 g (50% BWS), and 0.25 g (75% BWS). F1 and F2 denote the two main peaks of force. **(D)** Mean values of the first (F1) and second (F2) peaks derived from force records such as those of C plotted as a function of gravity level. Data of **(C,D)** are replotted from Ivanenko et al. (2002).

Gravity effects are essential for locomotion in contact with a support surface (Figure 1A). The downward force of gravity is resisted by the vertical ground reaction force (GRF), which a scale measures as body weight (mg) under static posture. When walking, the vertical GRF is time-varying, due to inertia (Figure 1B). Because of gravity, we must perform work on the COM at each step, even on a level surface (Cavagna et al., 2000). A full description of the mechanics of human locomotion is complex, given the great number of kinematic degrees of freedom and the even greater number of muscles that are involved at each step. Comprehensive analytical models defining the contributions of individual muscles, joints, and tendons are very difficult to be set up, and they may hide rather than reveal the general principles underlying the energetics of locomotion. By elaborating on the simplest possible mechanical model, instead, we can discern how the control of human locomotion takes advantage of the physical dynamics of bipedal gait (Kuo, 2002).

The simplest mechanical models of human locomotion involve two different oscillatory modes as a function of speed: the pendular mechanism of walking at low speeds and the bouncing mechanism of running at high speeds (Cavagna and Margaria, 1966; Mochon and McMahon, 1980; Full and Koditschek, 1999; Cavagna et al., 2000). Both mechanisms can be predicted theoretically by assuming that the specific gait mode is chosen based on energy optimization, and that energy cost is proportional to muscle work. In fact, these two mechanisms are selected—out of an infinite variety of different gait styles—by a computer model minimizing the mechanical cost of transport (Srinivasan and Ruina, 2006). At the top speeds of running, however, performance (push-average-power) rather than energy cost is minimized (Cavagna et al., 1991).

### Pendulum-like mechanism in walking

In an idealized fashion, walking is represented by an inverted pendulum under gravity (Figure 1A). The COM vaults along a circular arc about the supporting foot, slowing down as it rises, and speeding up as it falls. As a result, the changes of gravitational potential energy of the COM tend to be opposite to those of the corresponding forward kinetic energy, and mechanical energy is saved. A more realistic version of this model takes into account the fact that COM motion is only pendulum-like, and work needs to be performed to raise and lower the COM during single support, and to redirect the velocity vector of COM at the transition from one stance leg to the next (Cavagna et al., 1976; Neptune et al., 2004; Kuo et al., 2005). The maximum energy recovery due to the exchange of potential and kinetic energies is about 65% at the optimal speed of about 5.5 km/h at Earth gravity (Cavagna et al., 1983). Energy recovery is defined as the ratio between the external work saved by the pendulum-like mechanism and the maximum work that would occur without any energy exchange at the COM due to the pendulum mechanism. Recovery is maximum when the changes of gravitational potential energy are about the same amplitude and 180° out-of-phase relative to the changes of forward kinetic energy. Deviating substantially from a pendulum-like behavior can lead to significant increases of energy expenditure, as when people try to walk as level as possible by reducing vertical COM displacements (Massaad et al., 2007; Gordon et al., 2009). In addition to the inverted pendulum behavior of the stance limb, the contralateral limb simultaneously swings about the hip as an upright pendulum.

The pendulum-like features of walking are common to all individuals, but subjects differ in their ability to minimize energy oscillations of their body segments and to transfer mechanical energy between the trunk and the limbs (Bianchi et al., 1998a), based on a different tuning of intersegmental coordination (Bianchi et al., 1998b). Moreover, people in specific environments show an improved efficiency of the pendulum mechanism. Thus, African women from the Kikuyu and Luo tribes can carry heavy loads on their head without increasing the rate of metabolic energy consumption (Maloiy et al., 1986), and they do so by means of an improved pendulum-like transfer between gravitational potential energy and kinetic energy at the COM (Heglund et al., 1995; Cavagna et al., 2002).

### Pogo-stick mechanism in running

During running, the oscillations of the COM can be equated to those of a spring-mass system bouncing on the ground, as a bouncing ball or pogo-stick. In this case, gravitational potential energy and forward kinetic energy at the COM change in-phase (Cavagna et al., 1964). At each step the muscle–tendon units absorb and restore both the kinetic energy change of forward motion, due to the braking action of the ground, and the gravitational potential energy change, associated with the fall and the lift of the COM. This results in a large amount of negative and positive work and the chemical energy cost per unit distance is twice as large as that spent in walking at the optimal speed (Margaria, 1938). The metabolic energy expenditure is reduced in running, however, by an elastic storage and recovery of mechanical energy. In fact, when the leg strikes the ground, mechanical energy is temporarily stored as elastic strain energy in muscles, tendons, and ligaments and then it is partially recovered during the propulsive second half of the stance phase. Mechanical energy recovery in running has been estimated to be about 0.55 near the walk to run transition speed, and declining at higher speeds (Kaneko, 1990; Carr and Newman, 2007, 2017).

### Principle of dynamic similarity

The principle of dynamic similarity (Alexander, 1989) states that dynamically similar bodies have the same gait when the horizontal speed of COM *v* is normalized as the dimensionless Froude number: Froude = v^2^/gL, where L is the leg length and g is the acceleration of gravity. The Froude number is proportional to the ratio between kinetic energy and gravitational potential energy. It was originally proposed by William Froude (1874) to compare the hydrodynamic behavior of ships of very different sizes, and it was later applied to locomotion by Thompson (1917) and Alexander (1976), as well as others (see Vaughan and O'Malley, 2005). Thompson (1917) applied the Froude number to compare the theoretical stride length of the inhabitants of Lilliput and Brobdingnag, based on the heights of these people reported in Gulliver's Travels. Alexander (1976) used the Froude number to estimate the speeds of dinosaurs based on their footprints, and then generalized the regression of relative stride length and Froude number across several living quadrupedal mammals (Alexander and Jayes, 1983).

In theory, walking becomes impossible when the centrifugal force (mv^2^/L) exceeds the centripetal force due gravity (mg); above this limit, the body would take off in the aerial phase of running (Alexander, 1989). This corresponds to a Froude number greater than 1 (about 10 km/h for an average human at Earth gravity). The ratio of centrifugal to centripetal force constrains the walk-to-run transition to values of Froude number equal or less than 1, but does not specify a unique value. However, the theory of dynamic similarity (Alexander, 1989; Bullimore and Donelan, 2008) predicts a constant value of Froude number for the walk-to-run transition despite changes in g and L. In practice, on Earth, running becomes energetically more efficient (lower oxygen consumption) than walking at much lower speeds than the upper limit, namely speeds of about 7–8 km/h corresponding to a Froude number of about 0.5. It has also been found that a Froude number of about 0.25 corresponds to the optimal speed of walking (about 5 km/h), that is, the speed associated with the maximal recovery of mechanical energy (Cavagna et al., 1976; Saibene and Minetti, 2003).

Dynamic similarity implies that the recovery of mechanical energy in subjects of short height, such as children (Cavagna et al., 1983; DeJaeger et al., 2001; Ivanenko et al., 2004a), pygmies (Minetti et al., 1994), and people with dwarfism (Minetti et al., 2000), is not different from that of normal-sized adults when compared at the same Froude number. With respect to the present topic, dynamic similarity also implies that both the optimal walking speed and the walk-to-run transition speed depend on the gravitational level (Margaria and Cavagna, 1964). Indeed, the specific value of the acceleration of gravity *g* appears in the denominator of the ratio that defines the Froude number. Thus, the lower the gravity level, the lower will be the optimal walking speed and the walk-to-run transition speed (Saibene and Minetti, 2003). Moreover, we note that the limb geometry and the musculoskeletal apparatus contribute to the oscillatory behavior of the COM, and that general anatomy and individualized limb segment proportions are optimized in such a way that the Froude number can explain optimal walking speed. Indeed, when the relative proportion of lower limb segments is artificially changed by wearing stilts, one finds that the minimum metabolic cost per unit distance occurs at a lower Froude number than it does when walking without stilts (Leurs et al., 2011).

## Developmental considerations

We face gravity effects on the body as soon as we are born. Newborn babies exhibit a number of antigravity responses, such as the righting and the parachute reflexes. In addition, most human neonates can step automatically if supported under the armpits and placed in contact with a table. When stepping, they are able to support up to 40% of their body weight. They do so by generating alternating contractions of flexor and extensor muscles, which are controlled by spinal central pattern generators (CPGs) (Dominici et al., 2011; Ivanenko et al., 2013). Stepping mainly reflects spinal and brainstem control (Forssberg, 1985; Yang and Gorassini, 2006), as shown by the presence of the stepping reflex in anencephalic infants and infants with cervical spinal lesions (Peiper, 1961). Reflex stepping normally disappears around 1–2 months of age, but it can still be evoked during that period with daily practice (Zelazo et al., 1972; Yang et al., 1998) or supporting the weight of the legs by means of water immersion (Thelen and Cooke, 1987). Voluntary unsupported walking develops at 11–19 months, when infants have an improved postural control and weight support. However, many idiosyncratic features of infant stepping are not a simple result of postural instability (Forssberg, 1985; Ivanenko et al., 2005), and toddlers are still unable to fully compensate for body weight unloading (Dominici et al., 2007).

### Maturation of pendulum-like mechanism

**Figure 2:**
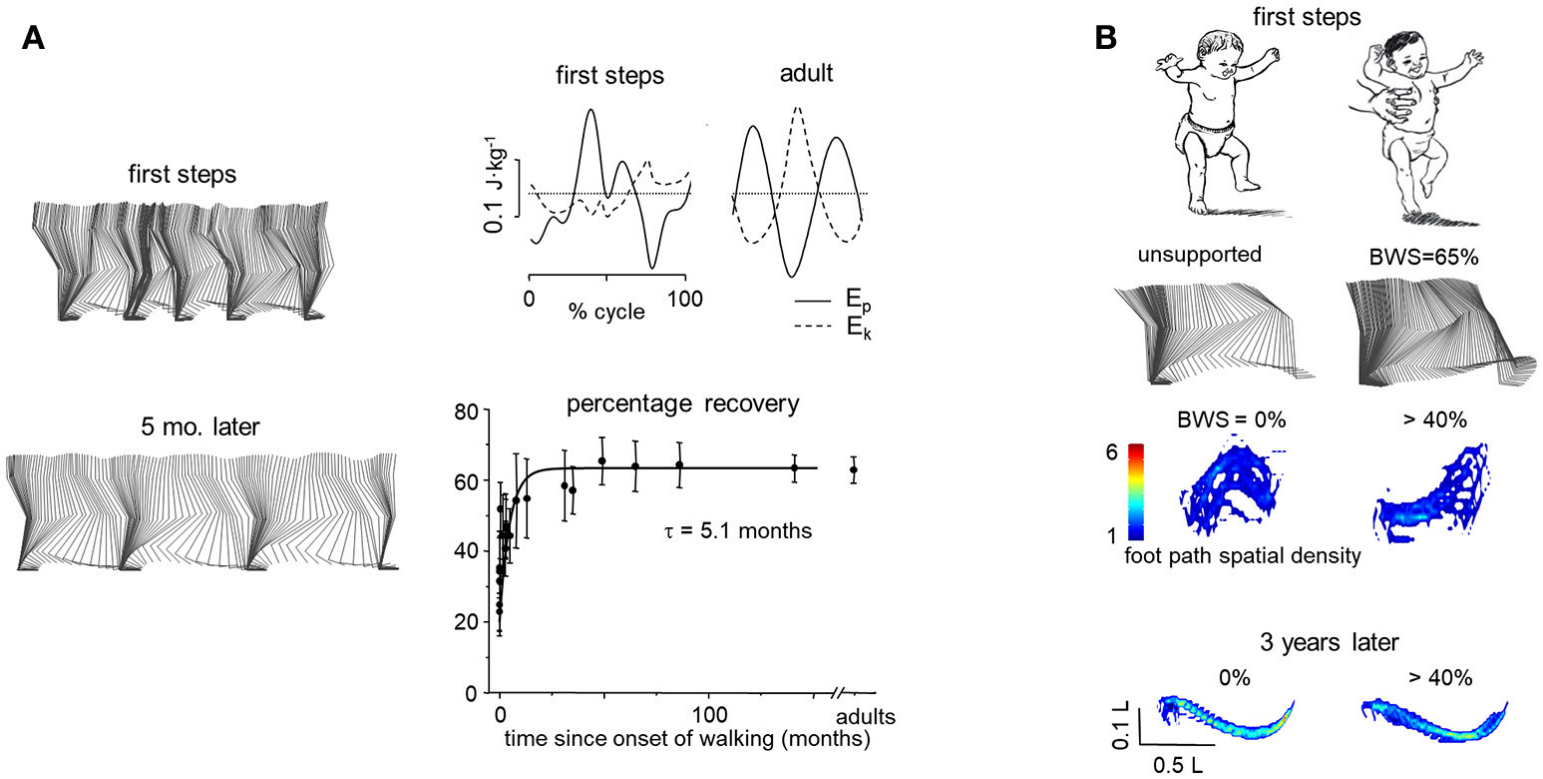
Developmental considerations. **(A)** examples of stick diagrams in the same child during first independent steps (12.5 mo) and 5 months later (left), changes of gravitational potential (E_p_) and kinetic energy (E_k_) of COM during a single stride in one toddler and one adult (top), and percentage of recovery of mechanical energy of COM as a function of the time after the onset of independent walking fitted by an exponential function (bottom) (adapted from Ivanenko et al., 2004a). **(B)** stick diagrams of 1 cycle and foot trajectory spatial density plots when the toddler walked unsupported (left) and unloaded (right). Spatial density of foot path was integrated over swing phase (across 10–15 steps) and depicted graphically by means of a color scale (adapted from Dominici et al., 2007): the lower the density (toward blue in color-cued scale), the greater the variability. Plots are anisotropic, vertical scale being expanded relative to horizontal scale. Bottom plots—the same child recorded 3 years later. Note a characteristic (one peak) foot path during normal walking in the toddler and changes in the shape of the foot trajectory when the child was unloaded.

Gravity-related signals during the first unsupported steps in toddlers contribute as a functional trigger for gait maturation (Ivanenko et al., 2004a, 2007). The pendulum-like mechanism of walking (Figure 2A) matures progressively in children, becoming established at a recovery percentage comparable to that of adults only around 2 years of age (Cavagna et al., 1983; Ivanenko et al., 2004a). In particular, in walking adults, the hip vaults over the stance leg as an inverted pendulum (Figure 1A) twice in each gait cycle, with two corresponding peaks in the temporal profile of vertical hip position. Instead, toddlers at the onset of unsupported locomotion show irregular and variable vertical trunk oscillations (Figure 2A, left panel). Accordingly, the changes in the potential and kinetic energies of the COM are also irregular, and the percentage of mechanical energy recovery is much lower than in adults (Figure 2A, right panels). After few months of walking experience, the vertical trunk oscillations become more regular and the recovery of mechanical energy of the COM approaches that of the adults (Figure 2A).

As noticed above, there are two pendular mechanisms during walking, the inverted pendulum of the stance limb pivoting around the foot and the upright pendulum of the contralateral limb that swings about the hip. Body weight support (or partially reduced gravity) noticeably affects the foot trajectory in toddlers, in contrast to adults and older children who show only limited changes in kinematic coordination under such conditions. Figure 2B illustrates the effects of the body weight support on the kinematics of the swing phase. When walking unsupported, toddlers demonstrate a characteristic one-peak foot path, while when they are unloaded the shape of the foot trajectory changes significantly. Toddlers tend to make a high foot lift and forward overshoot during the swing phase (Figure 2B). These findings suggest that, at the onset of independent walking in children, changes in gravitational loads on the body are not compensated accurately by the kinematic controllers. Instead, compensation and development of pendulum-like mechanism require several months of walking experience (Ivanenko et al., 2004a; Dominici et al., 2007). Thus, not only maturation of the neuromuscular system but also learning by experience represents a powerful optimizing process for a proper integration of the gravity-related limb and body dynamics of walking.

## Evolutionary and comparative considerations

### Some paleoanthropology

The pendulum-like behavior of walking is shared by many terrestrial animals with variable efficiency (Cavagna et al., 1977), but humans are the only extant animals who walk habitually with an erect, bipedal plantigrade posture. This style of locomotion represents a key adaptation of bipeds to gravity effects, since it yields the best alignment of the contact force vector with the lower limb joints and results in limited joint torques during stance (Biewener et al., 2004).

Further insights on its functional significance might be gained by considering the evolution of human bipedal locomotion (Bramble and Lieberman, 2004). Bipedal plantigrade walking appeared at least 4 million years ago (Leakey and Walker, 1997; Haile-Selassie, 2001; Vaughan, 2003; Zollikofer et al., 2005). It probably predated the evolution of large brains, and it may have acted as a co-factor favoring the expansion of the brain. Lucy's (A.L. 288-1) brain volume was similar to that of our closest cousins, the chimpanzees, amounting to only 1/3 the volume of the brain of modern humans, and with a larger cheek-tooth size than that of modern humans (McHenry, 1984). The earliest fossils that can be safely attributed to the hominin clade, the australopithecines like Lucy, resemble extant chimpanzees in numerous aspects of their locomotor skeleton, in addition to the skull (Tardieu et al., 2013). However, Lucy, as a member of Australopitecus afarensis species, probably walked as a habitual biped, whereas chimps typically walk quadrupedally. Lucy was much smaller (about 1–1.2 m high) than modern humans. Therefore, if she relied on pendulum-like walking (which we do not know), her optimal speed was correspondingly slower (as in children or pygmies). The evolution of bipedal locomotion can also be tracked by observing the transition in the footprints from australopithecus to homo erectus: only in the latter (about 1 My ago) do we find the modern features of adducted hallux, medial longitudinal arch, and medial weight transfer before push-off, while Australopitecus footprints are more similar to those of chimps (Bennett et al., 2009).

### Non-human primates

Chimpanzees are facultative bipeds in the wild, when they sporadically raise themselves on the hindlimbs. When they walk bipedally, their posture is much more flexed than that of modern humans. Despite being a bent-hip, bent-knee gait, bipedal chimpanzees walk with an inverted pendulum style of locomotion, with vertical oscillations of the COM that are similar in pattern but with a much lower efficiency than that of humans, the recovery percentage amounting to only about 15% (Demes et al., 2015). Notice that another primate closely related to chimpanzees, the bonobo, does not use an inverted pendulum (D'Août et al., 2004). When humans voluntarily adopt a knee- and hip-flexed posture while walking, the pendulum-like behavior is disrupted (Grasso et al., 2000), gravitational potential and kinetic energies fluctuating in-phase rather than out-of-phase as in normal erect walking (Li et al., 1996).

## Locomotion in actual hypogravity

### Moon walks

Five years before the first Moon walk, Margaria and Cavagna (1964) made the following theoretical predictions based on the inverted pendulum model: (1) walking would be possible only at very low speeds, (2) there should be a quick transition to running, (3) maximum running speed should be about 5 km/h, (4) jumping would be preferred at higher speeds. Indeed, assuming a Froude number of 0.5 for the walk-to-run transition, one would expect that it should occur around 3 km/h on the Moon (Minetti, 2001).

Anectodical reports and videos from Apollo 11-17 (1969-72) expeditions on the Moon roughly confirm Margaria and Cavagna predictions (Carr and McGee, 2009; Jones and Glover, 2013). There were a total of 14 Apollo expeditions with Moon walks from Apollo 11 (1969) to Apollo 17 (1972) involving 12 different astronauts, the first and the last astronaut to walk on the Moon being Neil Armstrong and Eugene Cernan, respectively. All EVAs (extravehicular activities, such as Moon walks) totaled about 78 h. Although there was considerable inter-subject variability in the preferred gait style (see Jones and Glover, 2013), the astronauts neither walked nor ran most of the time (also because of low frictional contact forces, which are proportional to gravity), but they mainly used one of three styles of locomotion: (1) loping, (2) skipping, and (3) hopping (see for instance https://www.youtube.com/watch?v=x2adl6LszcE). Loping is a kind of slow running with a high, long, prolonged aerial phase, and it is energetically optimal under reduced gravity conditions (Rader et al., 2007).

### Skipping

Skipping is especially interesting (Minetti, 1998), because it is a gait mode intermediate between walking and running, showing features of both the former (double support phase) and the latter (flight phase). Each foot undergoes two lift-off events in each cycle to produce a syncopated stepping. Skipping can be unilateral (right or left, depending on the last foot in contact with the ground prior to the flight), but is most commonly bilateral, with alternating right and left unilateral strides. The metabolic and biomechanical analyses of skipping (performed in the laboratory on Earth) show that the two basic strategies for mechanical energy saving of walking (pendulum-like) and running (elastic bouncing) are operating at different phases of the gait cycle (Minetti, 1998). Moreover, the timing of the major peaks of motoneuronal activity in the spinal cord resembles that of walking and running (Ivanenko et al., 2008a). Although little if ever used by human adults on Earth, skipping belongs to the repertoire of human gaits since childhood. Indeed it is a typically—though sporadically—performed by children at about 5 years of age. The most likely reason why skipping is later abandoned is that it is metabolically inefficient on Earth (Minetti, 1998).

### Role of space suit

When considering the Moon walks of astronauts, one should take into account that their weight was reduced by 1/6 (the ratio of Moon gravity to Earth gravity), but inertia was the same as on Earth. It is known that gravity interacts with inertia in complex ways, especially during running (Chang et al., 2000). Also the Apollo spacesuit worn by the astronauts affected locomotion. Although the mass of the space suit was about 80 kg, almost all of it was supported by the internal pressure forces (Carr, 2016). However, the space suit constrained the astronaut's movements considerably and involved significant metabolic costs, limiting the intensity and duration of EVAs.

Carr and McGee (2009) reviewed audio transcripts and video clips of lunar EVAs available from the Apollo Lunar Surface Journal (Jones and Glover, 2013), as well as from several NASA technical reports (cited in Carr and McGee, 2009). They identified gait events that could be classified as walk/lope/run and for which speed could be estimated. In their classification, the lope mode included skipping and hopping, in addition to loping. Using estimated speeds and individual anthropometric characteristics of each astronaut, they computed the Froude number for each such event. They found that the transition between walk/lope and run occurred at an average Froude number of 0.37, close to the value predicted by Margaria and Cavagna (1964) and by the dynamic similarity principle.

### Falls

Another element that characterizes locomotion on the Moon is the relative instability and propensity to fall. Indeed, falls or saved falls were frequent during the EVAs of Apollo missions (Kubis et al., 1972a,b; Bloomberg et al., 2016). In addition to external causes (e.g., slippery, loose or uneven terrain, low visibility, mobility constraints imposed by the spacesuit, see Scott-Pandorf et al., 2009), instability was often caused by inappropriate automatic postural reactions in response to tripping or stumbling. These reactions were often too fast and tended to facilitate rather than impede the fall (Kubis et al., 1972a,b; Bloomberg et al., 2016).

### Treadmill exercise on ISS

Nowadays, to mitigate the negative effects of prolonged weightlessness on the osteo-articular, muscular and cardiovascular systems, astronauts perform several exercises in the ISS, including walk and run on a treadmill (TVIS, Treadmill with Vibration Isolation Stabilization System). To this end, they wear a harness attached at the waist and shoulders with bungee cords that keep them in contact with the treadmill belt. The cords restore about 60% of the weight of a typical astronaut, and their pulling direction and force change with the movements. Therefore, the restoring forces are variable and are not tailored to each individual.

Recently, a novel Subject Loading System (SLS) has been designed for potential use on ISS (Gosseye et al., 2010). It consists of two pneumatic pistons attached at one end to the trunk harness and at the other end on a trolley sliding with the subject's movements. The resulting traction force is roughly equal to the weight of each subject and remains nearly constant, whatever the position of the subject on the treadmill. Laboratory tests on Earth found that the biomechanics of subjects running with the SLS was reasonably similar to that of normal running (Gosseye et al., 2010). The SLS might therefore represent a better tool for exercise on ISS.

## Postflight locomotion

Transitioning from one gravity level to another one is often troublesome. In particular, prolonged weightlessness affects post-flight human performance in several ways, and locomotion is no exception (Bloomberg et al., 2016). Upon return to Earth, crewmembers often experience ataxia, postural instability and navigation difficulties. One day after returning from an average flight duration of 185 days, astronauts completed the functional mobility test (an obstacle course over an unstable, compliant surface) in about twice the time used during the preflight test (Mulavara et al., 2010). Several specific parameters of gait were altered soon after long-duration space flight, and recovered to preflight values in a few days. Thus, knee flexion during stance was significantly increased (Bloomberg and Mulavara, 2003), angular motion at the knee and ankle and vertical accelerations of the COM were increased (Hernandez-Korwo et al., 1983), timing and magnitude of lower limb muscles were slightly but significantly altered, especially at heel strike and toe-off (Layne et al., 1997, 2004) in parallel with changes in toe-clearance (Miller et al., 2010).

## Simulated hypogravity with parabolic flight

Experiments in parabolic flight represent a viable alternative to spaceflight experiments; they are much less expensive, less demanding, and allow a larger sample of participants (Pletser, 2004; Shelhamer, 2016). Parabolic flight can simulate 0 g, 0.16 g (Moon), 0.4 g (Mars), or hyper-gravity (e.g., 1.5 or 2 g). The main drawback is represented by the limited time available to reproduce hypogravity conditions (~20, 30, and 40 s for 0, 0.16, and 0.4 g, respectively), which only allows for acute investigations, even though several parabolas are performed during a flight campaign. A few experiments on locomotion were carried out in parabolic flight, and the results depended on the level of simulated hypo-gravity.

### Simulating martian gravity

Cavagna et al. (1998, 2000) performed parabolic flights at 0.38 g. Their results were generally in agreement with the predictions of the inverted pendulum model and the principle of dynamic similarity. They found that the amplitude of energy changes (both gravitational potential energy and kinetic energy) was smaller at 0.38 g than at 1 g, and the step period was longer. A decreased work of walking at 0.38 g relative to 1 g is consistent with the decreased metabolic energy consumption reported during walking in reduced gravity Earth simulators (Newman et al., 1994). Maximum pendulum-like recovery of mechanical energy at COM was smaller than at 1 g (about 55 vs. 65%) and occurred at lower speeds (about 3.5 vs. 5.5 km/h). Thus, the range of walking speeds was about half as wide as on Earth, walk-to-run transition occurred at about the optimal speed of walking on Earth, and the external work performed to walk a given distance was about half as much as on Earth.

### Simulating moon gravity

De Witt et al. (2014) studied the walk-to-run transition at 0.16 g during parabolic flight. They found that participants preferred to run instead of walking at much higher Froude numbers than predicted by the inverted pendulum model and the dynamic similarity principle (on average, 1.4 instead of 0.5 Froude). They argued that deviations from the predicted Froude number are due to the accelerations induced by the swinging limbs that increase the downward force applied to the body by gravity, as previously suggested by Kram et al. (1997) based on simulated hypogravity on Earth (see below). Thus, if one replaced the term g in the Froude equation with the summed contribution of gravity and downward acceleration due to swinging limbs, one would account for the experimental values of walk-to-run transition speed in hypogravity (Kram et al., 1997; Raichlen et al., 2013). The protocol used by De Witt et al. (2014) to assess walk-to-run transitions during parabolic flight differs substantially from the protocols typically used on Earth. Due to the short parabola durations, participants judged the most comfortable speed among several constant treadmill speeds, instead of switching automatically as it occurs on Earth or Moon. However, these authors also performed the switching protocol in the Earth laboratory using a body weight unloading system and confirmed the results obtained with parabolic flights.

### Jumping and landing from a jump

Due to altered sensory information, altered anticipatory postural adjustments (Clément et al., 1984) and body reference configuration (Massion et al., 1997), reduced gravity conditions may also affect locomotor-related tasks. For instance, recent work on landing from a jump during parabolic flight demonstrated clear modifications in the preparatory adjustments and the loading phase, and suggested that otolithic information plays an important role in the control of landing from a jump (Gambelli et al., 2016a,b). In this work, pulldown forces were continuously generated by the SLS described above, simulating 1, 0.6, 0.4, and 0.2 g. Another recent paper studied jumping up on site with knee and hip joints almost extended during Lunar and Martian parabolas (Ritzmann et al., 2016). It found that the new gravitational load was anticipated correctly by the participants being compensated for by gravity-adjusted muscle activities.

## Simulated hypogravity on earth

### Gravity compensation devices

**Figure 3:**
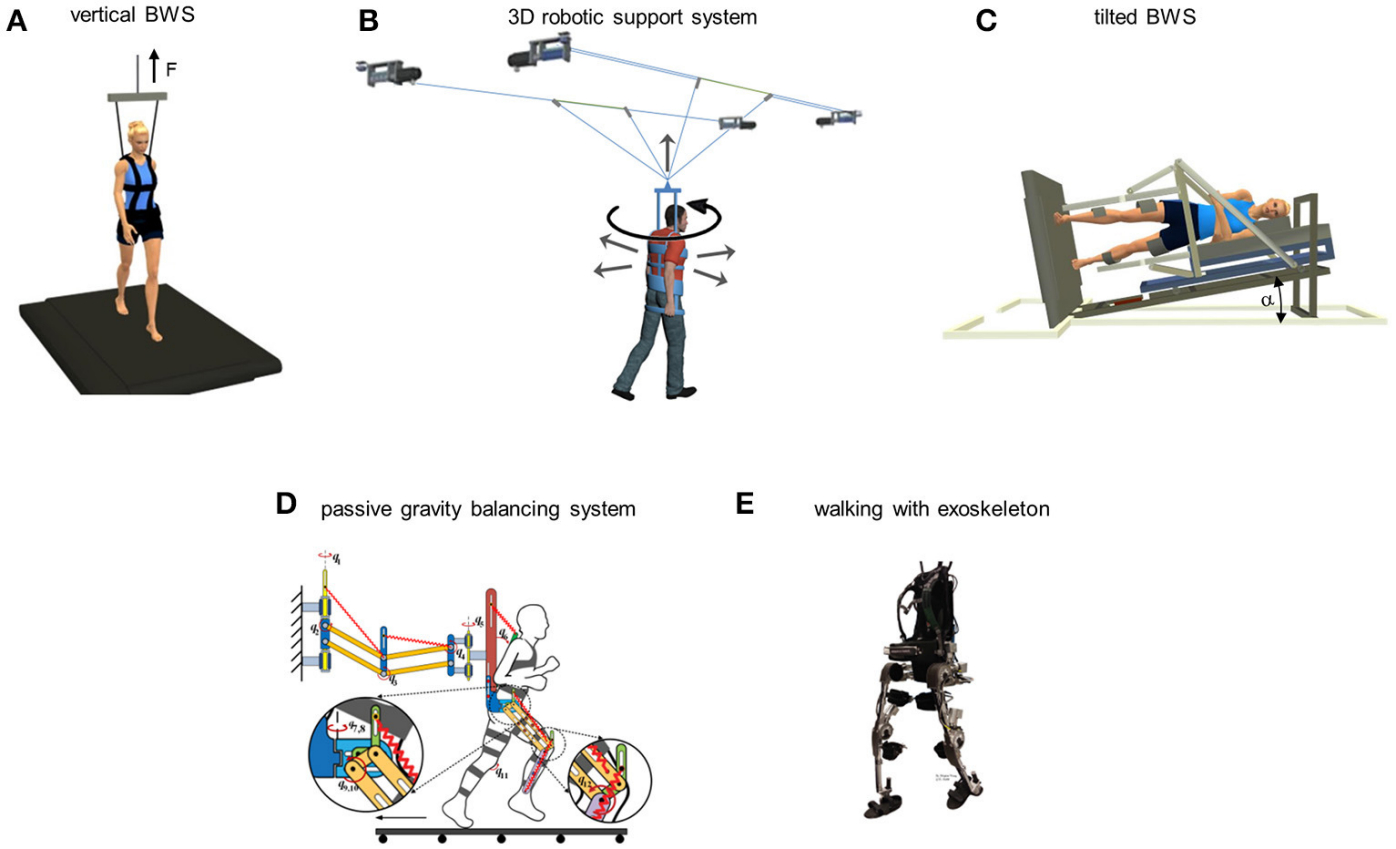
Different classes of reduced gravity simulators for locomotion. **(A)** vertical body weight support system: subject walks on a treadmill while being supported in a harness, pulled upwards by a preset unloading force F. **(B)** robotic support system that allows assisted 2D overground locomotion, including the directions of the actuated and passive (rotation) degrees of freedom (adapted from Mignardot et al., 2017). **(C)** tilted unloading system: subject lies on the side on a tilted couch with both legs suspended in the low friction exoskeleton and steps on the treadmill tilted to the same angle (adapted from Ivanenko et al., 2011). The component of the gravitational force acting on the stance and swing limbs is proportional to the tilting angle α. **(D)** passive reduced gravity walking simulator (courtesy of Dr. Ou Ma) consisting of the spring-balanced dual-parallelogram mechanism to compensate for the weight of the body and the legs. **(E)** Mindwalker exoskeleton for assisted walking in spinal cord injured individuals (adapted from Wang et al., 2015).

Obviously gravity cannot be changed on Earth, but there exist several simulators that allow low-cost, long duration trials of reduced gravity conditions. Each of these simulators has advantages and disadvantages, but all of them assist posture and locomotion by countering the force of gravity on the body. One of the more frequently used simulators is the body weight support (BWS) system for walking on a treadmill. In vertical BWS, the subject is supported in a harness that applies a controlled upward force to the trunk (Figure 3A). Mignardot et al. (2017) modified a ceiling-mounted system to apply multidirectional forces to the trunk (Figure 3B), based on the observation that vertically restricted trunk support alters gait dynamics and that the addition of well-calibrated forward forces alleviates these effects. Most available BWS systems constrain locomotion to a treadmill, or a restricted path for vertical unloading systems that are suspended on a shifting cart. Awai et al. (2017) developed a ceiling-mounted rail and deflector system that allows unrestricted walking on level ground or on stairs.

A different approach to counteract the force of gravity in the vertical position consists in applying lower-body positive pressure (LBPP), increasing air pressure around the lower body to create a lifting force approximately at the COM (Cutuk et al., 2006; Grabowski and Kram, 2008; Ruckstuhl et al., 2009; Grabowski, 2010; Schlabs et al., 2013). LBPP systems allow ambulation in the normal erect posture, but high pressure levels may affect systemic blood pressure, head perfusion, and vascular flow, thus requiring caution (Cutuk et al., 2006). Moreover, LBPP provides the desired vertical weight support, but may also generate unwanted horizontal assistance due to the interface between the chamber and the subject (Grabowski and Kram, 2008).

The main limitations of all these simulators are given by the high pressure localized to specific regions of the skin (at the harness attachment for vertical BWS or the body parts within the pressurized chamber for LBPP), and the application of the vertical unloading force only to the trunk, so that during stance the lower limb experiences a reduction of effective gravity force proportional to the unloading force. However, these systems cannot aid the swing movements of the limbs, because they do not pull them in proportion to the simulated gravity level and therefore the limbs remain subject to full gravity during swing.

The latter disadvantage is overcome by the tilted BWS systems (Ivanenko et al., 2011) or horizontal suspension systems (Gurfinkel et al., 1998; De Witt et al., 2010). These simulators do not allow a normal posture, but do not involve major cardiovascular risks. They have been used by both Roscosmos and NASA to train astronauts prior to the mission (Hewes, 1969; Bogdanov et al., 1971; Hansen, 1995). NASA, in particular, has several facilities for horizontal suspension (e.g., the Langley's Reduced Gravity Walking Simulator or the Enhanced Zero-gravity Locomotion Simulator). Tilting the subject counteracts a fraction of body weight, the component of the gravity force acting on the body and limbs in the sagittal plane of walking being reduced in proportion to the cosine of the tilt angle relative to the horizontal. One such system (Italian patent number Rm2007A000489, Figure 3C) involves a bed supporting the head, trunk, and upper limbs, while the lower limbs are suspended in low-friction, low-mass exoskeletons (Ivanenko et al., 2011; Sylos-Labini et al., 2011, 2013). The subject lies on the side, while the bed and exoskeletons can be tilted by an angle between 0° and 40°. The subject steps on a treadmill which is tilted by the same angle. In contrast with the vertical BWS, tilted BWS simulators provide a comfortable lying position for the subject and reduce the effects of gravity on both stance and swinging limbs. As a drawback, however, they involve the extra mass of the moving couch and exoskeleton, and limit lateral trunk movements while walking. Another system is represented by the passive gravity balancing system (Figure 3D). It is anchored to a wall and compensates the weight of the body by means of a spring-balanced, dual-parallelogram mechanism, and torso-support assembly, while the weight of each leg is compensated by a leg exoskeleton (Lu et al., 2011; Ma and Wang, 2012). Partial gravity replacement loads can be applied by means of pneumatic pistons with the SLS or similar systems while the subject is in weightlessness (Gambelli et al., 2016a,b) or horizontally tilted in the Earth laboratory (Ivanenko et al., 2011).

Several passive or active gravity compensation devices have been developed in robotics to allow human subjects to step freely over-ground (see Arakelian, 2016). The first exoskeletons were designed to reduce the burden for soldiers and help them carrying heavy objects (e.g., Walsh et al., 2006; Zoss et al., 2006). Subsequently, exoskeletons have been used in rehabilitation, for instance the Indego, EKSO Bionics, ReWalk, Mindwalker (Figure 3E) (del-Ama et al., 2012; Esquenazi et al., 2012; Wang et al., 2015). Some exoskeletons do not allow any voluntary contribution from the subject, while others include an assist-as-needed control principle, which is beneficial to learn stepping. The MoonWalker is a passive force balancer that sustains body-weight (Krut et al., 2010). It is controlled using an actuator requiring very low energy on flat terrains to shift the force on the stance leg. The actuator can also provide part of the energy to climb stairs or slopes. van Dijk et al. (2011) developed a passive exoskeleton to minimize joint work during walking. This exoskeleton uses artificial tendons, acting in parallel with the leg. Artificial tendons are elastic elements that are able to store and redistribute energy over the human leg joints. In contrast with BWS systems, exoskeletons apply the unloading force to the feet rather than the trunk. Therefore, the exoskeleton provides full postural stability and does not necessitate antigravity muscle activity from the subject, but the feet still experience the full subject's weight during walking.

### Biomechanical effects of simulated reduction of gravity

**Figure 4:**
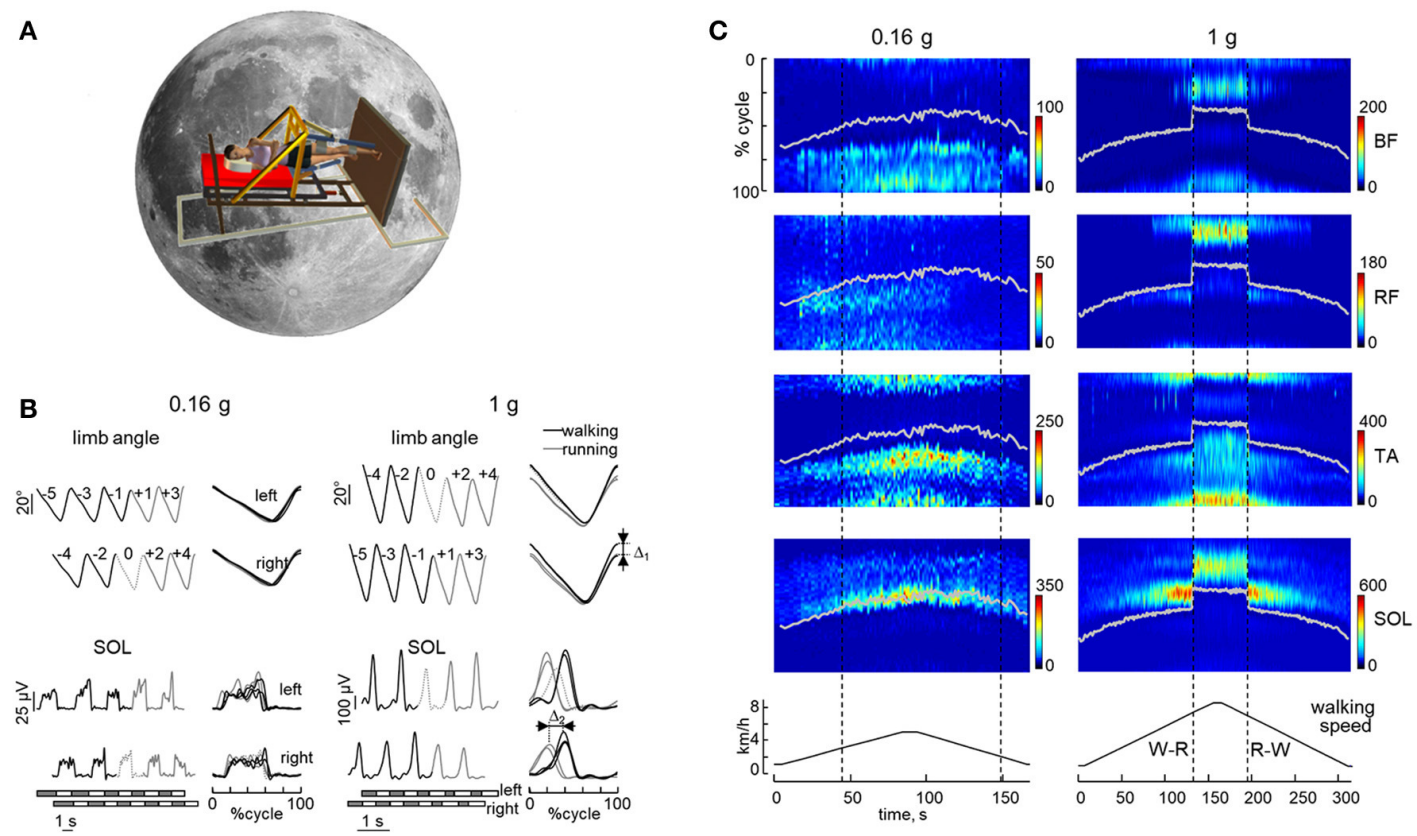
Abruptness of gait transitions on Earth (1 g) and smoothness of gait transitions at reduced simulated gravity (0.16 g) during slow changes in treadmill belt speed. **(A)** simulation of Moon (0.16 g) gravity was obtained by using the tilted BWS system (Figure 3C). **(B)** limb angle and soleus EMG waveforms (left, plotted vs. time; right, plotted vs. normalized cycle) during five consecutive strides of both legs around the transition from walking (black lines) to running (gray lines). Dotted curves denote the transition stride (stride 0, in which the swing phase first exceeded 50% gait cycle). Bottom horizontal bars denote swing (white) and stance (black) phases. Δ1 and Δ2—abrupt changes in the limb angle and the timing of the SOL activity peak at gait transition at 1 g (between stride +1 and stride −1). **(C)** example of EMG patterns during slow changes in treadmill speed. The color maps represent a sequence of discrete activation waveforms (vertical slices): x-axis indicates the number of the gait cycles (corresponding to the appropriate timing of the trial), y-axis indicates normalized gait cycle, and color indicates EMG amplitude. The white line indicates when toe off occurred. Vertical dashed lines indicate walk-to-run (W-R) and run-to-walk (R-W) transitions. RF, rectus femoris; BF, biceps femoris; TA, tibialis anterior; SOL, soleus. Adapted from Sylos-Labini et al. (2011).

Simulations of hypogravity on Earth using different BWS techniques generally confirmed the basic principles of gravity effects on locomotion (for a review, see Sylos-Labini et al., 2014b). In particular, they confirmed that reduced gravity involves lower optimal walking speeds and lower preferred walk-to-run transition speeds as compared with normal gravity. However, significant deviations from the Froude numbers predicted by the dynamic similarity principle have been reported (Donelan and Kram, 1997, 2000; Kram et al., 1997; De Witt et al., 2014). Thus, Ivanenko et al. (2011) and Sylos-Labini et al. (2013) compared locomotion at 0.07 g (simulating Pluto), 0.16, 0.38, and 1 g, using the vertical and the tilted BWS, or the 0.16 g replacement load in the recumbent position. Consistent with previous observations (Donelan and Kram, 1997; Kram et al., 1997; De Witt et al., 2014), they found that walk-to-run transitions occur at Froude > 0.5 at simulated gravity < 0.2 g. They also found a hysteresis in gait transitions (walk-to-run transition speed >> run-to-walk transition speed) at these low gravity levels. More interestingly, they found that gait transitions at reduced gravity were gradual (Figure 4), without any noticeable abrupt change as it typically occurs at 1 g, for both kinematics and muscle activity patterns (Ivanenko et al., 2011; Sylos-Labini et al., 2011). Figure 4 illustrates an example of abrupt gait transition on Earth (1 g) and smooth walk-to-run transition at simulated Moon gravity (0.16 g) in a representative subject. For instance, the percent stance duration, the limb angle at foot contact and the timing of leg muscle EMG activity were all significantly different between the stride immediately before (stride −1) and immediately after (stride +1) the walk-to-run transition at 1 g, but they were not significantly different at reduced gravity (Figures 4B,C). It should be noticed that some gait parameters (horizontal foot excursion, maximum horizontal foot speed, relative swing duration) depended on the type of BWS, vertical vs. tilted (Sylos-Labini et al., 2013), suggesting an important contribution of the swing limb dynamics, which is affected differentially by the specific apparatus.

### Kinematics

**Figure 5:**
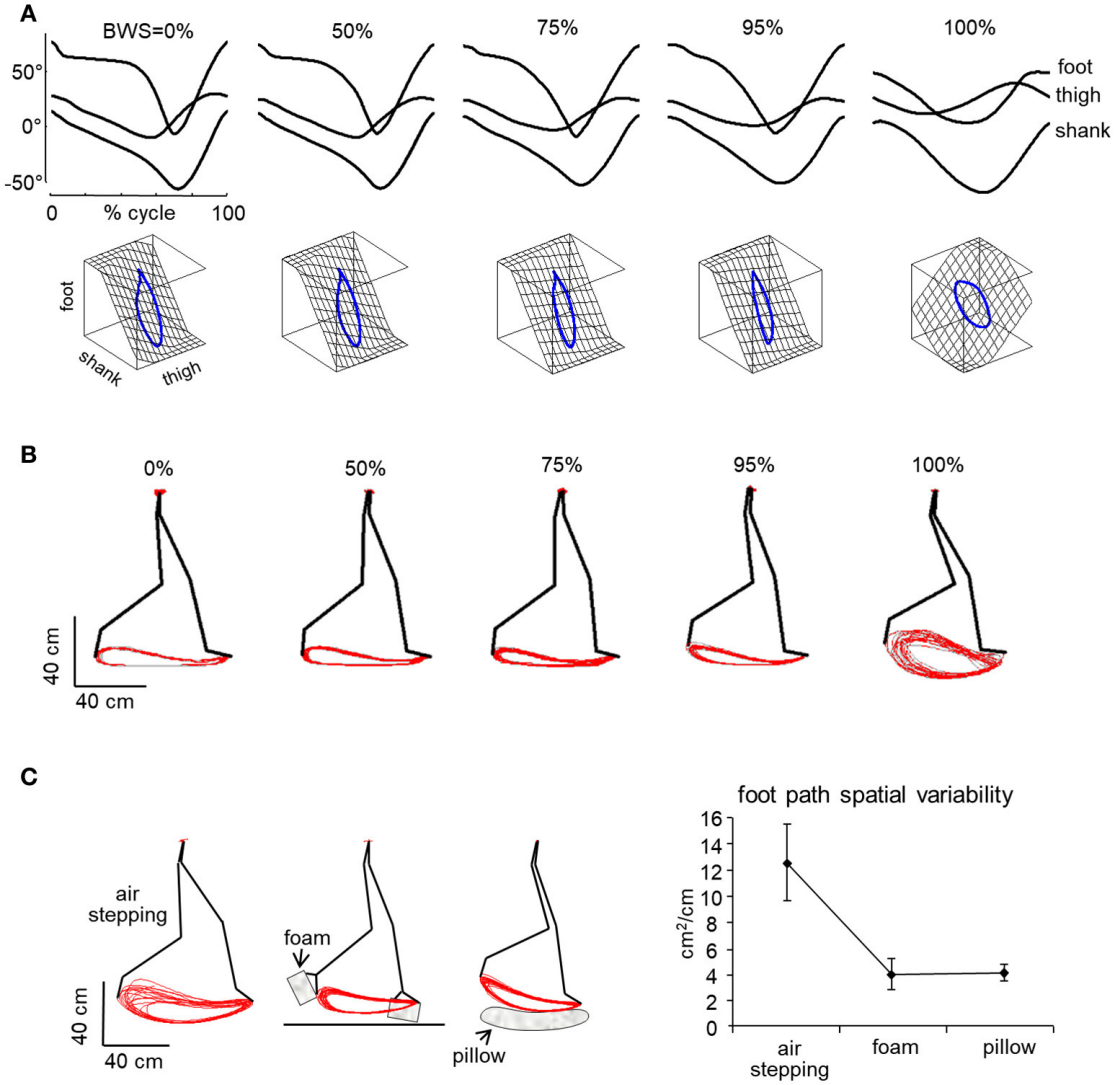
Effect of simulated reduced gravity on walking kinematics. **(A)** examples of ensemble-averaged elevation angles of the lower limb segments in one adult subject walking at 3 km/h at different BWS levels (100% refers to air-stepping) and corresponding 3-dimensional gait loops obtained by plotting the elevation angles 1 vs. the others. Note similar kinematics and covariance plane orientation in all BWS conditions except for air-stepping. Adapted from Ivanenko et al. (2002). **(B)** shape and variability of endpoint path in 1 subject over 12 consecutive step cycles for trials performed at different BWS levels. **(C)** effect of minimal contact forces provided by a foam-rubber taped under the subjects' feet or when touching the pillow during air-stepping. Right panel—spatial variability of the foot trajectory (foot path tolerance area, mean ± *SD* over all subjects). Note that the shape and variability of the foot path is comparable in all conditions except in air-stepping, where variability is much higher **(B)**. Variability decreased substantially in the presence of minimal surrogate contact forces relative to standard air-stepping **(C)**.

**Figure 6:**
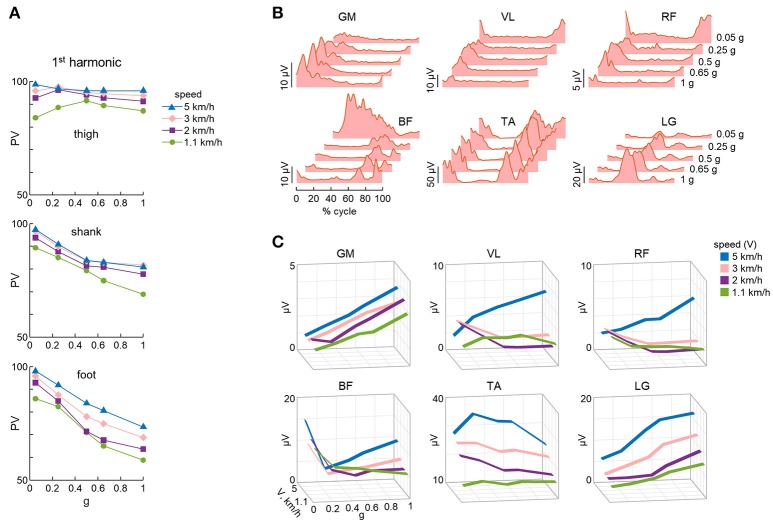
Non-linear changes of kinematic and muscle activity patterns. **(A)** Percent variance (PV) (mean values over all trials and subjects) accounted for by the 1st harmonic of each elevation angle (see Figure 4A) as a function of BWS. **(B)** EMG activity of lower limb muscles for a participant walking at 3 km/h at different simulated reduced gravity levels. **(C)** Mean EMG activity computed over the gait cycle and averaged across all subjects (*n* = 8), as a function of speed and simulated reduced gravity. GM, gluteus maximus; VL, vastus lateralis; RF, rectus femoris; BF, biceps femoris; TA, tibialis anterior; LG, lateral gastrocnemius. Data are replotted from Ivanenko et al. (2002).

The more detailed studies with vertical BWS also showed limited changes of the kinematic coordination across a wide range of gravity levels (0, 0.05, 0.25, 0.5, 0.65, 1 g), despite drastic changes of the kinetic parameters (Ivanenko et al., 2002). Thus, the peak vertical contact forces decreased proportionally to the unloading force (Figures 1C,D), so that at 0.05 g they were 20-fold smaller than at 1 g and were applied at the forefoot only instead of showing the classical heel to ball shift. By contrast, the trajectory of the feet in space and the planar covariance of limb segment elevation angles were very similar at all gravity levels except for 0 g, when subjects could only step in air (Figures 5A,B). Indeed, the 3D gait loops obtained by plotting the elevation angles one vs. the others lied close to a plane during walking (denoted by grids in Figure 5A). Planar co-variation was documented for different walking conditions (Bianchi et al., 1998a; Grasso et al., 2000; Ivanenko et al., 2008b) and it was obeyed at all tested speeds and BWS levels (Ivanenko et al., 2002). However, the specific phase relationships between the elevation angles were different during air-stepping, leading to a different plane orientation at 100% BWS in Figure 5A. In addition, there were some non-linear changes in the waveforms of the limb segment elevation angles, for instance, the contribution of the 1st harmonic to the shank and foot elevation angles became more prominent with reduced gravity level (Figure 6A).

In the absence of support–related constraints (air-stepping), both inter-stride and inter-subject variability were considerably augmented (Ivanenko et al., 2002, 2008b). On the other hand, minimal contact forces were generated artificially during air-stepping by taping a foam-rubber under the subjects' feet that lightly touched the belt of the treadmill during the stance phase or by having the subject stepping on a pillow (Figure 5C, left). Such minimal contact forces were sufficient to significantly decrease the variability of the foot path (Figure 5C, right), suggesting that the support surface represents an importance reference frame for accurate foot trajectory control (Ivanenko et al., 2002). These results might be relevant for the special conditions of moving around and working on a small asteroid with very low gravity (<10^−3^ g, Garrick-Bethell and Carr, 2007).

### EMG patterns

Also the analysis of the electromyographic patterns (EMG) revealed a non-linear trend with the gravity level (Ivanenko et al., 2002, 2004b, 2013; Van Hedel et al., 2006; Klarner et al., 2010; Sylos-Labini et al., 2014b; Fischer et al., 2015). The temporal components shared by multiple muscles during walking with the vertical BWS were very similar across the tested gravity levels between 1 and 0.05 g (see above), but the weighting coefficient of each component on individual muscles differed considerably as a function of the gravity level (Ivanenko et al., 2004b). Moreover, the amplitude of net activity of most muscles did not scale proportionally to the percent of body weight loading (Figure 6B), as did the amplitude of the peak vertical contact force (Ivanenko et al., 2002). For some muscles, the changes with loading were not even monotonic, and there was a complex reorganization of the pattern of activity which differed across individuals, evidence of variable adaptive adjustments to reduced gravity (Sylos-Labini et al., 2014b). For instance, the mean amplitude of activity in ankle extensors decreased systematically with decreasing simulated gravity, consistent with their antigravity function (lateral gastrocnemius muscle, see LG in Figure 6C). By contrast, the activity of rectus femoris muscle showed increments at slow speeds (<3 km/h, Figure 6C), while the hamstring muscles demonstrated new activity bursts with body weight unloading (Ivanenko et al., 2002). The latter muscles are those showing the largest variability across subjects (Ivanenko et al., 2002; Sylos-Labini et al., 2014a). Overall, the changes in muscle activity depend on the changed biomechanical requirements with BWS, changed inertial or assistive forces, complex architecture of skeletal muscles, and the dynamic coupling of limb segment motion (Zajac et al., 2003). Even the activity of synergistic muscles, such as gastrocnemius and soleus muscles, may show substantial differential changes during decreased limb loading (Ivanenko et al., 2002; McGowan et al., 2010). In addition, apparently paradoxical elevated EMGs during overground walking assisted by an exoskeleton compared to normal walking have been reported in healthy subjects, consistent with an important contribution of foot loading-related sensory feedback (Sylos-Labini et al., 2014a). These findings indicate that the control algorithms for robotic assistance still need to be optimized, for instance by being tailored to each individual (Mignardot et al., 2017). There might also be the need for extensive training sessions of the individuals wearing the exoskeleton, so that they become fully adapted to assisted locomotion.

### Body weight unloading in toddlers

There are still few studies on the physiological effects of reduced gravity in children, although this is an important topic given the growing application of BWS techniques in pediatric rehabilitation (see next section). One study (Dominici et al., 2007) compared the locomotion under partial weight unloading in toddlers (about 1 year old) at their first unsupported steps, older children (1.3–5 years), and adults. To simulate various levels of body weight in a manner acceptable by a child, an experimenter held the trunk of the child with both hands and supplied an approximately constant vertical force during stepping on a force platform. In contrast to adults and older children who showed only limited changes in kinematic coordination under reduced-gravity (see above), toddlers lifted the feet too high and forward at the end of swing (Figure 2B, Dominici et al., 2007). Intermediate walkers (1.5–5 mo after walking onset) showed only partial improvements in foot trajectory characteristics. Therefore, at the onset of walking, changes in vertical body loads are not compensated fully by the CNS (Forssberg, 1985; Ivanenko et al., 2005; Dominici et al., 2007).

## Clinical applications of simulated hypogravity

BWS locomotion training has shown some promise as a tool to facilitate locomotor activity in individuals with neuromotor disorders, such as spinal cord injury SCI (Hubli and Dietz, 2013), stroke (Sale et al., 2012; Mehrholz et al., 2014; Moraru and Onose, 2014), Parkinson disease (Miyai et al., 2000; Picelli et al., 2013), Multiple Sclerosis (Swinnen et al., 2014), Cerebral Palsy, and Down syndrome (Damiano and DeJong, 2009; Valentin-Gudiol et al., 2013). However, in some patients, the efficacy of BWS-treadmill interventions is limited (Morawietz and Moffat, 2013; Picelli et al., 2013). In particular, this intervention seems to have weak or conflicting evidence in children with Cerebral Palsy (Dewar et al., 2015). On the other hand, it may accelerate the development of independent walking in children with Down syndrome (Damiano and DeJong, 2009; Valentin-Gudiol et al., 2013). Stroke patients who are able to walk, but not those unable to walk, benefit from the intervention by increasing walking speed and endurance (Mehrholz et al., 2014), although the superiority of the intervention relative to other control therapies has failed to be established (Charalambous et al., 2013). In SCI patients, locomotor training may induce the reappearance of kinematic regularities (Grasso et al., 2004a), EMG temporal components shared by multiple muscles (Ivanenko et al., 2003), and flexor reflexes (Smith et al., 2014). Robot-assisted training improves mobility-related outcomes to a greater degree than conventional over-ground training for patients with incomplete SCI, particularly during the acute stage (Nam et al., 2017). A low rather than a high testing treadmill speed may be beneficial for an optimal expression of EMG improvements in individual with incomplete chronic SCI (Meyns et al., 2014). A critical combination of sensory cues might be required to generate and improve locomotor patterns after SCI during assisted locomotor training (Hubli and Dietz, 2013). Mignardot et al. (2017) recently developed an adaptive algorithm that personalizes multidirectional forces applied to the trunk based on patient-specific motor deficits. This multidirectional gravity-assist enabled natural walking in individuals with SCI or stroke.

In spite of a complex non-linear reorganization of muscle activity patterns with BWS (Ivanenko et al., 2002; Moreno et al., 2013), the basic spatiotemporal structure of the locomotor output tends to be preserved in healthy and SCI subjects (see above), implying that a few oscillating circuits drive the active muscles to produce locomotion (Gerasimenko et al., 2010, 2015). These characteristic spatiotemporal features of spinal motorneuron activation are becoming increasingly important also for the functional assessment and rehabilitation of walking after SCI, both in SCI patients using BWS-treadmill training (Ivanenko et al., 2003; Grasso et al., 2004a), or in animal models of SCI, e.g., in robotic rehabilitation in spinalized rats with epidural stimulations mimicking physiological timings of muscle activations (Capogrosso et al., 2016; Wenger et al., 2016).

Several aspects need to be taken into consideration when using BWS locomotor training to restore the locomotor function. First, the term “normal motor pattern” is somewhat misleading under hypogravity conditions. As noticed above, there are considerable non-linear changes in the muscle activity patterns with changing BWS, especially in proximal leg muscles, even in neurologically intact individuals (Ivanenko et al., 2002; Moreno et al., 2013). It has also been recommended that very low speeds and high levels of BWS, where the EMG differences are most prominent, should be avoided whenever possible in the rehabilitation practice (Van Kammen et al., 2014). Second, the current assessments of therapeutical efficacy of body weight unloading in gait pathologies should consider the complex nature of the control of locomotion, task-dependent features, individual compensatory strategies, and plasticity of neuronal networks. For instance, locomotor training with BWS in SCI patients may not generalize to untrained walking conditions. Thus, SCI patients (ASIA-A, B, and C) were trained to step on a treadmill with BWS for 1.5–3 months (Grasso et al., 2004b). At the end of training, foot motion recovered the shape and the step-by-step reproducibility that characterize normal gait. The patients were then asked to step backward on the treadmill belt moving in the opposite direction. In contrast to healthy subjects who can immediately reverse the direction of walking by time-reversing the kinematic waveforms, all tested patients were unable to step backward initially and they needed specific training in the new direction (Grasso et al., 2004b).

In sum, BWS has contributed importantly to the methodologies that can be used to restore the locomotor function in disabled people. BWS systems are now often used for gait rehabilitation to assist locomotor recovery by performing well-focused and carefully directed repetitive practice. It is also worth noting the beneficial effect of simulated weightlessness on rhythmogenesis and its potential for assessing the state of the spinal pattern generation circuitry and for developing CPG-modulating treatments (Ivanenko et al., 2017). As a final point, an effective strategy to stimulate the spinal cord circuitries and to promote neuroplasticity in disabled people is a combination of locomotor training in simulated hypogravity with other promising experimental approaches, such as epidural electrical stimulation or drug application (Hubli and Dietz, 2013; Angeli et al., 2014; Minassian et al., 2016; Gad et al., 2017; Shah and Lavrov, 2017).

## Conclusions and perspectives

Our knowledge on the adaptation of locomotion to hypogravity has progressed considerably over the last few years, but remains fragmentary. There are still too few observations in actual hypogravity (Moon and parabolic flight), while most quantitative data come from laboratory simulations. The latter provide only partial replicates of true hypogravity because of the constraints imposed by physics. Indeed, when the locomotor kinematics, EMG, and ground reaction forces are compared between parabolic flight and horizontal suspension on Earth, subtle but systematic differences are revealed (De Witt et al., 2010). Even different simulators on Earth may yield slightly but significantly different results between each other, in terms of both locomotor kinematics and EMG patterns (Ivanenko et al., 2011; Sylos-Labini et al., 2011, 2013, 2014b). The limitations of ground experiments become even more evident when compared with the reports about locomotion on the Moon, where the specific characteristics of the environment (dust, uneven terrain, thin atmosphere, wide range of temperatures, heavy, and cumbersome spacesuits, etc) play an important role. Laboratory experiments with locomotion on soft (Lejeune et al., 1998) or complex terrain (Matthis et al., 2017) are therefore highly relevant.

Training crew members prior to space missions is quite challenging, and it remains particularly difficult to simulate the effects of reduced forces on all physiological systems and apparatus. Thus, prior to their missions, the Apollo crews had limited parabolic flight exposure and mainly trained in the Lunar Landing Training Vehicle, which did not simulate the vestibular effects of 0.16 g. Indeed, upon return after the mission, most Apollo astronauts reported having felt “wobbly” on the lunar surface initially, but that their coordination improved steadily during the first few hours of lunar EVA (Bloomberg et al., 2016). Unsteadiness is even more severe upon re-entry to Earth from a long-term space mission, because the gravity replacement systems used on the ISS to exercise do not act on the whole body, and in particular do not act on the vestibular system. It thus seems imperative to be able to design appropriate training protocols and exercise countermeasures with available technologies, to train space travelers to become more adaptable to transitions in gravity levels, such as from a higher to a lower gravity or vice versa. In the future, rotating habitat units capable of generating artificial partial gravity might become a valid countermeasure to physiological deconditioning during long duration space missions (such as in the Mars direct project, Clément et al., 2016).

One important lesson we learnt from a wealth of studies, including those reviewed here, is that a reduction of gravity does not lead necessarily to linear, continuous changes of locomotor parameters. Departures from linearity may take the form of non-proportional changes of a given locomotor parameter as a function of the gravity level, or we may observe a discontinuity with the transition to a qualitatively distinct behavior. Some such behaviors are predictable based on theory. For instance, according to the dynamic similarity principle, both the optimal walking speed and the walk-to-run transition speed scale with the square root of gravity (Alexander, 1989). Also, the transition from walking to skipping at relatively low speed at Moon gravity can be predicted based on energy optimization criteria (Minetti, 1998; Ackermann and Van den Bogert, 2012; Pavei et al., 2015). Finally, at reduced gravities (Moon gravity or lower), humans could theoretically run on water, an impossible task on Earth. This latter performance was predicted by hydrodynamic modeling, and demonstrated using vertical BWS in the laboratory (Minetti et al., 2012).

However, other non-linear behaviors are discovered empirically, without necessarily being predicted by available theories. Thus, the activity of several limb muscles was shown to scale non-linearly with simulated hypogravity and differentially as a function of the individual muscles (Ivanenko et al., 2002; Sylos-Labini et al., 2011, 2014b). Moreover, several gait parameters change abruptly in the transition from walk to run at 1 g, but they change smoothly, gradually at lower gravities (Ivanenko et al., 2011). Also, below a given (still undefined) gravity threshold, foot contact forces become insufficient to drive the normal kinematic and EMG patterns of locomotion, and the CPGs switch to a different control mode (Ivanenko et al., 2002).

Another lesson stemming from recent studies is the potential usefulness of reduced gravity simulators for clinical applications on Earth. Our brief review has shown that the results are still mixed, possibly because of our incomplete understanding of the physiology of locomotor adaptation to reduced gravity and/or the inadequate methodologies employed to reduce gravity effects on the patient. One take-home message that is shared by the field of space travel and clinical rehabilitation is that individual data about the physiology and medical history of each person will likely contribute to reducing health and mission risks in space travelers and improving the outcomes of rehabilitation in patients. In particular, both reduced gravity simulators and training protocols should be tailored to each individual (White and Averner, 2001; Mignardot et al., 2017), rather than being stereotyped as it still is in the majority of cases.

## Author contributions

All authors listed have made a substantial, direct and intellectual contribution to the work, and approved it for publication.

### Conflict of interest statement

The authors declare that the research was conducted in the absence of any commercial or financial relationships that could be construed as a potential conflict of interest.
